# Dietary *Lycium barbarum* Polysaccharide Induces Nrf2/ARE Pathway and Ameliorates Insulin Resistance Induced by High-Fat via Activation of PI3K/AKT Signaling

**DOI:** 10.1155/2014/145641

**Published:** 2014-06-22

**Authors:** Yi Yang, Wang Li, Yan Li, Qing Wang, Ling Gao, Jiajun Zhao

**Affiliations:** ^1^Department of Endocrinology, Provincial Hospital Affiliated to Shandong University, Jinan 250021, China; ^2^Key Laboratory of Fertility Preservation and Maintenance of Ministry of Education, Ningxia Medical University, Yinchuan 750004, China; ^3^Biochemistry and Molecular Biology, Ningxia Medical University, Yinchuan 750004, China; ^4^Central Laboratory, Provincial Hospital Affiliated to Shandong University, Jinan 250021, China; ^5^Institute of Endocrinology, Shandong Academy of Clinical Medicine, Jinan 250021, China

## Abstract

*Lycium barbarum* polysaccharide (LBP), an antioxidant from *wolfberry*, displays the antioxidative and anti-inflammatory effects on experimental models of insulin resistance *in vivo*. However, the effective mechanism of LBP on high-fat diet-induced insulin resistance is still unknown. The objective of the study was to investigate the mechanism involved in LBP-mediated phosphatidylinositol 3-kinase (PI3K)/AKT/Nrf2 axis against high-fat-induced insulin resistance. HepG2 cells were incubated with LBP for 12 hrs in the presence of palmitate. C57BL/6J mice were fed a high-fat diet supplemented with LBP for 24 weeks. We analyzed the expression of nuclear factor-E2-related factor 2 (Nrf2), Jun N-terminal kinases (JNK), and glycogen synthase kinase 3*β* (GSK3*β*) involved in insulin signaling pathway *in vivo* and *in vitro*. *First*, LBP significantly induced phosphorylation of Nrf2 through PI3K/AKT signaling. *Second*, LBP obviously increased detoxification and antioxidant enzymes expression and reduced reactive oxygen species (ROS) levels via PI3K/AKT/Nrf2 axis. *Third*, LBP also regulated phosphorylation levels of GSK3*β* and JNK through PI3K/AKT signaling. *Finally*, LBP significantly reversed glycolytic and gluconeogenic genes expression via the activation of Nrf2-mediated cytoprotective effects. In summary, LBP is novel antioxidant against insulin resistance induced by high-fat diet via activation of PI3K/AKT/Nrf2 pathway.

## 1. Introduction

Chronic oxidative stress, characterized by the overproduction of reactive oxygen species (ROS) [1–3], is associated with glucose and lipid metabolic abnormalities [4–6]. High glucose concentrations and fatty acid levels stimulate excessive accumulation of ROS, which can cause the deleterious effects [7–9] and insulin resistance in peripheral metabolic tissues [10, 11] in the absence or presence of antioxidants circumstance. Overall, oxidative stress represents an imbalance between production of ROS and the antioxidant defense system.

The mechanistic link between increased ROS and insulin resistance is activation of several signaling pathways. Nuclear factor E2-related factor 2 (Nrf2) is important to explore the role of the endogenous antioxidant system in the prevention of insulin insensitivity* in vivo*, which regulates the expression of detoxifying and antioxidant genes, such as heme oxygenase 1 (HO-1), superoxide dismutase (SOD), and catalase (CAT) [12, 13]. In response to oxidative stress or pharmacological activation, Nrf2 is translocated into the nucleus and induces the expression of antioxidant enzymes by binding antioxidant response element (ARE) [14–16]. Jun N-terminal kinases (JNK) activation is a crucial mediator of ROS-induced insulin resistance [17]. Suppression of JNK activation prevents insulin receptor substrate-1 (IRS-1) degradation and promotes insulin signaling and insulin-dependent glucose uptake [18].

Long-term high-fat diet (HFD) aggravates the burden of antioxidative and anti-inflammatory system in the liver [6]. Impairment of endogenous redox system of liver is important in the development of insulin resistance in chronic HFD feeding [19]. In our study, we detected Nrf2 signaling pathway* in vivo* and* in vitro*. Administration of* Lycium barbarum* polysaccharide (LBP), a new PI3K/AKT/Nrf2 axis activator, prevented the development not only of oxidative stress but also of insulin resistance, as well as of glucose metabolic abnormalities [20–22]. Activated Nrf2 of LBP represents a potential novel approach in the treatment and prevention of insulin resistance induced by HFD.

## 2. Results

### 2.1. LBP Ameliorates Insulin Resistance Induced by HFD in C57BL/6J Mice

**Figure 1:**
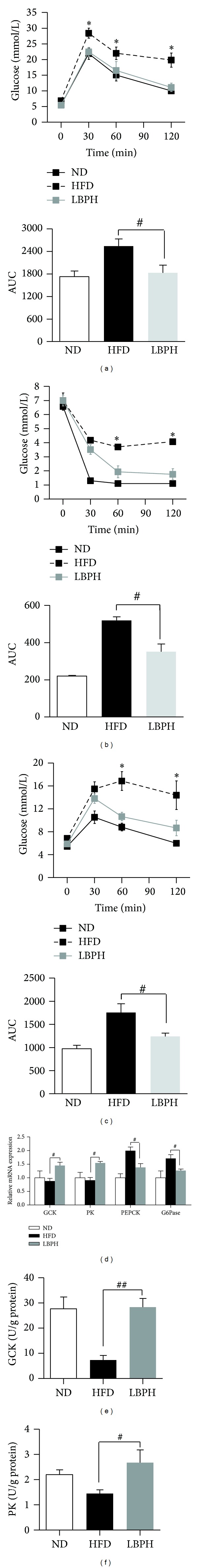
LBP prevents HFD-induced insulin resistance and modulates glucose metabolism* in vivo*. Injected intraperitoneal (a) glucose tolerance test, (b) insulin tolerance test, and (c) pyruvate tolerance test. Down: area under the curve (AUC). All data were expressed as means ± SEM (*n* = 6 per group), ^#^ *P* < 0.05. (d) The mRNA levels of GCK, PK, PEPCK, and G6Pase were analyzed by qRT-PCR. Data was expressed as means ± SEM (*n* = 3 per group), ^#^ *P* < 0.05. (e) GCK and (f) PK activities were measured with enzymatic colorimetric activity kits. Data was expressed as means ± SEM (*n* = 6 per group), ^#^ *P* < 0.05. LBPH, 100 mg/kg LBP plus high-fat diet.

To determine whether LBP reduces HFD-induced insulin resistance, glucose, insulin, and pyruvate tolerance tests were measured. Intraperitoneal glucose tolerance test (IPGTT) showed that LBP (100 mg/kg) significantly reduced blood glucose 30 min after injection; area under the curve (AUC) reflected the point (Figure 1(a), *P* < 0.05). As shown in Figure 1(b), intraperitoneal insulin tolerance test (IPITT) showed that LBP improved insulin-mediated glucose-lowering 60 min after injection; AUC also reflected the point (*P* < 0.05). In Figure 1(c), intraperitoneal pyruvate tolerance test (IPPTT) showed that LBP lowered blood glucose 60 min after injection; AUC also indicated the point (*P* < 0.05). On 24 weeks, we, respectively, measured blood glucose, insulin, and pyruvate concentrations in serum of different groups. As shown in Table 1, LBP reduced blood glucose and insulin concentrations (*P* < 0.05, *P* < 0.01) and increased pyruvate concentration compared to HFD-fed mice (*P* < 0.05).

### 2.2. LBP Modulates Glycolytic and Gluconeogenic Genes Expression in Liver of HFD-Fed Mice

In order to get a first insight into a potential interdependence between LBP and glucose metabolism, we analyzed glycolytic and gluconeogenesis genes expression levels with qRT-PCR. As shown in Figure 1(d), LBP increased GCK and PK mRNA levels and also decreased PEPCK and G6Pase mRNA levels (*P* < 0.05). LBP obviously elevated GCK and PK activities (Figures 1(e) and 1(f), *P* < 0.05, *P* < 0.01).

### 2.3. LBP Activates PI3K/AKT Signaling Pathway* In Vitro* and* In Vivo*

**Figure 2:**
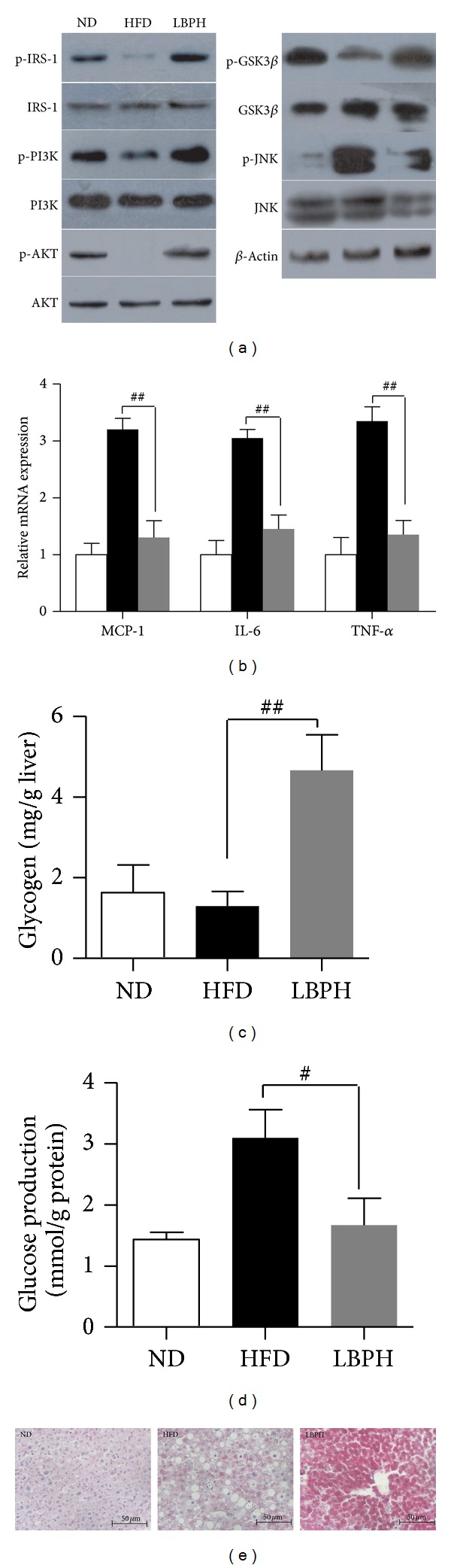
LBP suppresses HFD-induced inflammation and regulates glucose metabolism in liver. (a) Western blot analyzed phospho-IRS-1, -PI3K, -AKT, -JNK1/2, and -GSK3*β* levels. Representative western blots are shown. (b) qRT-PCR analysis of MCP-1, IL-6, and TNF-*α* in liver of LBP-treated and untreated mice. Data was expressed as means ± SEM (*n* = 3 per group), ^##^ *P* < 0.01. (c) Glycogen concentrations of liver (mg/g liver). (d) Glucose production of liver (mmol/g protein). Data was expressed as means ± SEM (*n* = 6–8 per group), ^#^ *P* < 0.05, ^ ##^ *P* < 0.01. (e) Representative periodic acid-schiff (PAS) staining on liver (400x).

We examined the effects of LBP on PI3K/AKT signaling pathway* in vivo* and* in vitro*. Treatment of LBP significantly enhanced the phosphorylation expression of IRS-1, PI3K, and AKT in liver (Figure 2(a)). HepG2 cells were incubated for 12 hrs with LBP in the presence of 200 *μ*M palmitate. From 100 *μ*g/mL to 600 *μ*g/mL concentration, LBP significantly promoted an increase of phospho-IRS-1, -PI3K, and -AKT levels (Figure 4(a)).

### 2.4. LBP Regulates Phosphorylation Levels of GSK3*β* and JNK via PI3K/AKT Signaling Pathway

In* in vivo* experiment, treatment of LBP effectively inhibited phospho-JNK level and increased phospho-GSK3*β* level in liver of HFD-fed mice (Figure 2(a)). The mRNA levels of inflammatory genes were analyzed by qRT-PCR in liver. As shown in Figure 2(b), LBP lowered expressions of MCP-1, IL-6, and TNF-*α* (*P* < 0.01). In Figures 2(c) and 2(e), LBP significantly promoted glycogen synthesis and glucose utilization (*P* < 0.01). In addition, LBP significantly reduced hepatic glucose production (HGP) in liver (Figure 2(d), *P* < 0.05). In* in vitro* experiment, cells were pretreated with 100–600 *μ*g/mL LBP for 12 hrs in the presence of palmitate. LBP significantly increased phosphorylation level of GSK3*β* and reduced phosphorylation level of JNK depending on dose-concentration (Figure 4(a)). In Figures 4(c) and 4(d), LBP also increased glycogen contents and decreased glucose production of palmitate-stimulated cells (*P* < 0.05, *P* < 0.01). When cells were pretreated for 2 hrs with 10 *μ*M LY294002 and 2 *μ*M Wortmannin of PI3K/AKT inhibitor, respectively, and then treated for 12 hrs with 300 *μ*g/mL LBP, we found that inhibitor-induced phospho-JNK level was suppressed by LBP, and inhibitor-suppressed phospho-GSK3*β* level was reversed by LBP (Figure 4(b)). Based on these findings, we conclude that LBP-supplement boosts cellular glycogen metabolism through activation of PI3K/AKT and JNK signaling pathway.

### 2.5. LBP Induces Nrf2 Phosphorylation Level* In Vivo* and* In Vitro*

**Figure 3:**
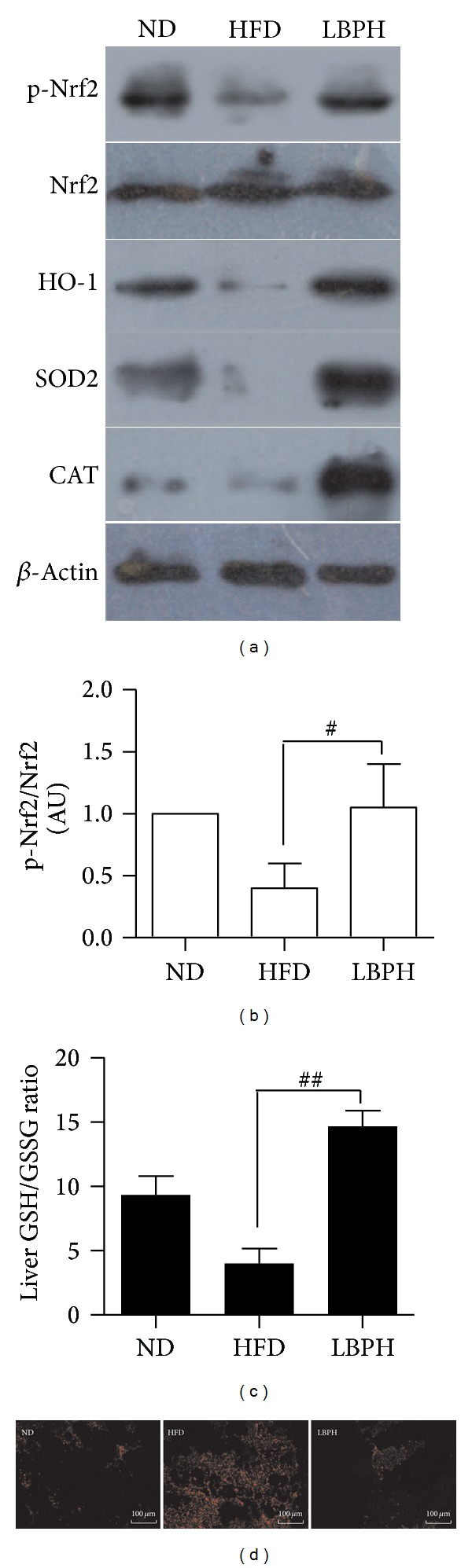
LBP induces Nrf2 phosphorylation and activates Nrf2/ARE pathway* in vivo*. (a) Immunoblotting analysis of phospho-Nrf2, total Nrf2, HO-1, SOD2, and CAT in liver. Representative western blots are shown. (b) The ratio of phospho-Nrf2/Nrf2. Data was normalized to the control and expressed as means ± SEM (*n* = 3), ^#^ *P* < 0.05. (c) The ratio of GSH/GSSG of liver. Data was expressed as means ± SEM (*n* = 6–8 per group), ^#^ *P* < 0.05. (d) Intracellular ROS levels of frozen liver sections (200x).

**Figure 4:**
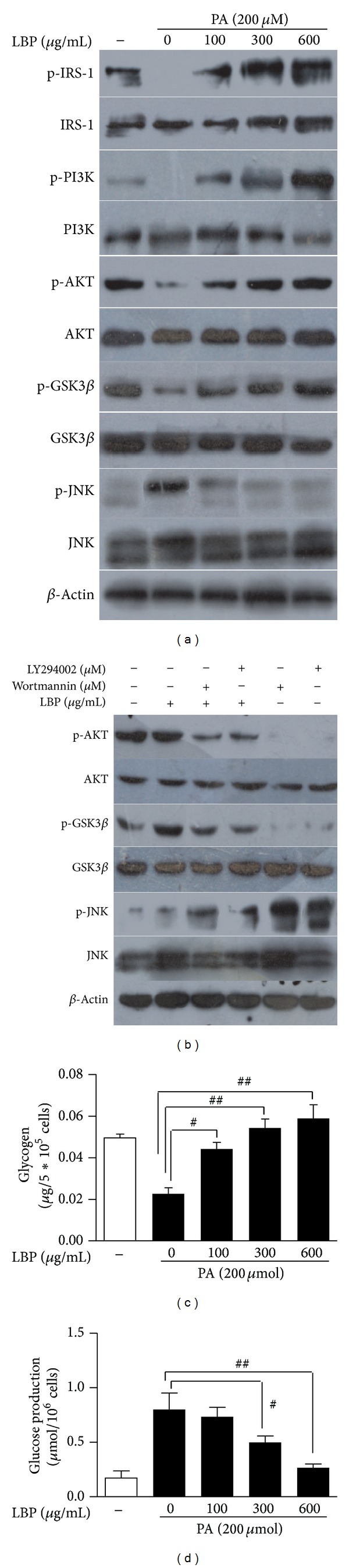
LBP improves the phosphorylation levels of JNK and GSK3*β* via regulation of PI3K/AKT signaling. (a) 200 *μ*M palmitate-stimulated HepG2 cells were treated with LBP for 12 hrs in dose-dependent manner. Immunoblotting analysis of LBP-mediated phosphorylation levels of IRS-1, PI3K, AKT, JNK1/2, and GSK3*β*. (b) Cells were treated with 10 *μ*M LY294002 for 2 hrs and 2 *μ*M Wortmannin for 2 hrs and then incubated for 12 hrs with 300 *μ*g/mL LBP. Immunoblotting analyzed phosphorylation of LBP-mediated AKT, JNK, and GSK3*β*. Representative western blots are shown. (c) Glycogen concentrations (*μ*g/5 × 10^5^cells) and (d) glucose production (*μ*mol/10^6^ cells) of LBP-treated cells in the presence of palmitate. Data was expressed as means ± SEM (*n* = 5), ^#^ *P* < 0.05, ^##^ *P* < 0.01.

**Figure 5:**
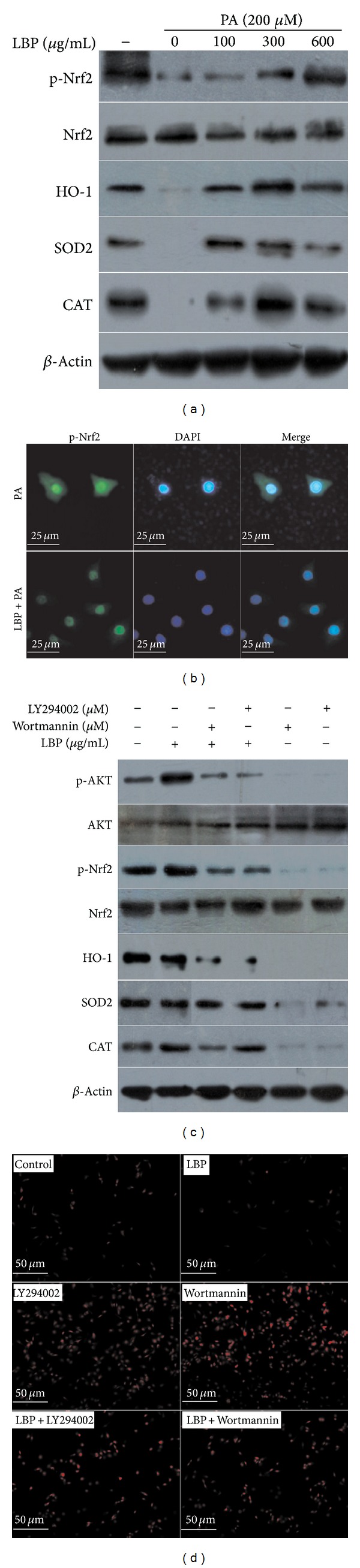
LBP increases Nrf2 phosphorylation and induces Nrf2/ARE pathway via activation of PI3K/AKT signaling. (a) 200 *μ*M palmitate-stimulated HepG2 cells were treated with LBP for 12 hrs in dose-dependent manner. Phospho-Nrf2, total Nrf2, HO-1, SOD2, and CAT were detected by western blot analysis. (b) 300 *μ*g/mL LBP-treated and untreated cells in the presence of 200 *μ*M palmitate. Representative images of phospho-Nrf2 localization (400x). (c) Cells were treated with 10 *μ*M LY294002 for 2 hrs and 2 *μ*M Wortmannin for 2 hrs and then incubated for 12 hrs with 300 *μ*g/mL LBP. Immunoblotting analyzed LBP-mediated AKT, Nrf2 phosphorylation, HO-1, SOD2, and CAT. Representative western blots are shown. (d) Cells were treated as following previous described and then incubated for 1 h with 50 *μ*M DHE. Representative images of intracellular ROS level (200x).

To test whether LBP activates Nrf2 expression* in vivo*, we delivered LBP at 100 mg/kg by intragastric administration to C57BL/6J mice for one day, respectively. We found that LBP could activate phospho-Nrf2 level, without any change in total Nrf2 protein expression (Figures 3(a) and 3(b), *P* < 0.05). We also examined whether LBP directly induced Nrf2 expression* in vitro*. Palmitate-stimulated cells were incubated in LBP for 12 hrs. As shown in Figure 5(a), LBP significantly increased phospho-Nrf2 expression in a dose-dependent manner, without any change in total Nrf2 expression. To investigate the detailed mechanisms underlying the induction of Nrf2 expression by LBP, we explored the nuclear translocation of Nrf2 with immunofluorescence experiments. 200 *μ*M palmitate obviously resulted in phospho-Nrf2 migrating from nucleus to cytosol, while 300 *μ*g/mL LBP caused the nuclear translocation of phospho-Nrf2 (Figure 5(b)).

### 2.6. LBP Activates Nrf2/ARE Pathway via PI3K/AKT Signaling

To further elucidate, the effects of LBP on Nrf2/ARE pathway were examined. LBP induced HO-1, SOD2, and CAT protein expression in liver of HFD-fed mice (Figure 3(a)). LBP also increased SOD and CAT activities in liver (Table 1, *P* < 0.01). As shown in Figure 5(a), LBP significantly increased the protein expression of HO-1, SOD2, and CAT in a dose-dependent manner in the palmitate-stimulated environment. In Table 1 and Figure 3(c), LBP displayed an increase in liver GSH and GSH/GSSG levels, which was significantly higher than HFD-fed mice by 24 weeks. However, GSSG levels were higher in the HFD-fed group compared with LBP-supplementation group (*P* < 0.05, *P* < 0.01). LBP significantly decreased intracellular ROS level (Figure 3(d)). Cells were pretreated with inhibitor and then treated with LBP. These results showed that LBP significantly activated Nrf2/ARE pathway in the presence of inhibitor (Figure 5(c)). It was an important point that inhibitor-induced intracellular ROS levels were reversed by 300 *μ*g/mL LBP (Figure 5(d)). Taken together, our data suggests that LBP induces phosphorylation level of Nrf2 and activates Nrf2/ARE pathway via PI3K/AKT signaling.

## 3. Discussion

Oxidative stress is a major factor in the development of various liver diseases, affects liver function and induces hepatic insulin resistance, and is ultimately attributed to liver injury [23, 24]. It is essential to control the progressive oxidative stress to upregulate cellular redox system [25]. The previous study has already reported that plant drugs reduce oxidative stress to maintain peripheral insulin sensitivity through increasing antioxidant or anti-inflammation effects [26–28].* Lycium barbarum* (*L. barbarum*) (*Gouqizi*,* Wolfberry*), whose bioactive components are* Lycium barbarum* polysaccharide (LBP), is well known in traditional Chinese herbal medicine. Previous studies have shown that LBP can protect liver function and reduce blood glucose levels [29–31]. But the effect of LBP on Nrf2-mediated insulin resistance is still not clear. We made a hypothesis in our study that investigated molecular mechanism underlying the activation of PI3K/AKT/Nrf2 signaling pathway by LBP treatment.

The discovery of Nrf2 as main factor in the expression of endogenous antioxidant genes favored study exploiting Nrf2 as target of drugs or nutritional supplements [27, 32, 33]. In the present study, we found that LBP supplementation can affect status of Nrf2 expression* in vivo* and* in vitro*. First, our data suggested that treatment of LBP can increase the phosphorylation level of Nrf2 in liver of HFD-fed mice. Second, our results showed that LBP effectively increased the phospho-Nrf2 expression in dose-dependent manner in palmitate-induced HepG2 cells. Third, inhibitor-suppressed Nrf2 expression was activated by LBP. Consistent with previous studies, it was shown that drugs had a positively antioxidative effect on animal models via activation of PI3K/AKT/Nrf2 pathway [34, 35].* In vitro* and* in vivo* results finally indicated that LBP as a new Nrf2 activator directly induced Nrf2 expression. Nrf2 is essential for cellular protective mechanisms against oxidative stress or inflammation through the transcriptional activation of ARE-dependent expressions of genes encoding detoxification enzymes and antioxidant enzymes such as HO-1, SOD2, CAT, and GSH [12, 36–39]. A previous study revealed that Nrf2-deficiency reduced the expression of detoxification enzymes in liver, resulting in oxidative stress [40, 41]. Antioxidant enzymes can improve insulin resistance in* in vivo* study [42]. Our results suggested that LBP effectively upregulated HO-1, SOD2, and CAT expression and reduced intracellular ROS levels through Nrf2 activity* in vivo* and* in vitro*. These results indicated that activation of Nrf2 is central to the induction of potent cellular antioxidant and detoxification systems.

JNK is a crucial mediator of insulin resistance, activated by the accumulation of ROS [43, 44]. Our results suggested that HFD-induced phospho-JNK was increased in liver. In contrast, treatment of LBP significantly reduced JNK phosphorylation level and expression of inflammatory genes, such as MCP-1, IL-6, and TNF-*α*. We further confirmed that LBP prevented expression of inflammatory factor by HFD-induced via PI3K/AKT/JNK pathway. Taken together, LBP, as an inhibitor of JNK, results in marked improvement of insulin sensitivity in mouse models of HFD-induced insulin resistance.

Activation of transcription factor Nrf2 is one of the major cellular defense lines against oxidative stress but also influences genes involved in glucose metabolism [32]. We investigated the effects of LBP on glucose homeostasis in HFD-fed mice for 24 weeks. We found that treatment with LBP increased phospho-GSK3*β* level and glycolytic enzymes expression but decreased gluconeogenic enzymes expression. In* in vitro* experiment, LBP also significantly increased hepatic glucose uptake and export through Nrf2 and JNK signaling. In summary, these studies demonstrate a critical role for Nrf2 in protecting the liver from stress likely by coordinately regulating expression of genes in cytoprotective and metabolic pathways.

In conclusion, our results provide a link between Nrf2 activity, oxidative stress, and insulin resistance and demonstrate* in vivo* and* in vitro* that high-fat induced-insulin resistance could be ameliorated by LBP through upregulating PI3K/AKT/Nrf2 signaling pathway. The current study suggests that LBP may be a promising role for managing insulin resistance-associated oxidative stress in acute or chronic liver damage.

## 4. Materials and Methods

### 4.1. Preparation of* Lycium barbarum* Polysaccharide

LBP was extracted from* L. barbarum* as previously described [45]. Briefly, the dried fruit of* L. barbarum* was put in boiling deionized water. The water extract was filtered through a filter paper to remove impurities. The crude extract was concentrated to the volume under vacuum at 40°C and diluted to deionized water. Then, the extract was precipitated with 95% ethanol, followed by centrifugation to remove the supernatant. Then, the precipitate was collected and ground into powder. The powder of LBP was dissolved in normal saline for mice experiment, filtered through a 0.22 *μ*m filter, and stored at −20°C.

### 4.2. Animal Care and Treatment

Male C57BL/6J mice from Beijing Vital River Biological Co., Ltd., were housed in standard cages placed in a room at 22 ± 2°C temperature, 55 ± 1% relative humidity, and a 12/12 light/dark cycle. All mice consumed a commercial diet and tap water for 2 weeks prior to their division into three groups (*n* = 10 per group): ND (10% energy from fat, D12450B, USA), HFD (60% energy from fat, D12492, USA), and 100 mg/kg LBP-supplemented diet. At the end of the animal experiments in 24 weeks, liver tissue was isolated, one sample was prepared for RNA isolation and analysis of gene expression, and another sample was frozen in liquid nitrogen and stored at −80°C. The animal experiments were approved by the Animal Research Committee of Ningxia Medical University, China.

### 4.3. Cell Culture and Treatment

HepG2 cells were generously provided from Peking University and cultured in DMEM medium (Sigma) supplemented with 10% fetal bovine serum (Gibco, USA) in 5% CO_2_ at 37°C. The cells were incubated in 200 *μ*M palmitate (Sigma) for 12 hrs and then treated with 100–600 *μ*g/mL LBP for 12 hrs. In addition, the cells were pretreated with 10 *μ*M LY294002 for 2 hrs (Cell Signaling Technology) and 2 *μ*M Wortmannin (Sigma) for 2 hrs and treated with 300 *μ*g/mL LBP for 12 hrs.

### 4.4. Glucose, Insulin, and Pyruvate Tolerance Tests

Mice (*n* = 6 per group) were fasted overnight for tolerance tests prior to testing. Following the fasting, glucose (2 g/kg), insulin (0.75 IU/kg), or pyruvate (2 g/kg) was injected intraperitoneally at 0 time. Blood samples were measured with glucometer (Accu-Chek, Roche Diagnostics) from tail vein at 0, 30, 60, and 120 min.

### 4.5. Biochemical Analysis

In collected blood and liver tissue from mice for 24 weeks, serum was centrifuged at 3,000 r.p.m for 15 min at 4°C. Serum insulin and pyruvate were measured with mouse ELISA kit (CUSABIO). Liver GSH and GSSG levels were measured using enzymatic colorimetric assay (BIOXYTECH, Portland, OR) according to the manufacturer's instructions.

### 4.6. Enzyme Activity Assays

Liver superoxide dismutase (SOD) and catalase (CAT) activities were determined by enzymatic colorimetric activity kits (NanJing Jiancheng Bioengineering Institute, China) according to the manufacture's instruction. Liver glucokinase (GCK) and pyruvate kinase (PK) activities were measured with the Assay Kit (NanJing Jiancheng Bioengineering Institute, China) according to the manufacture's instruction.

### 4.7. Glycogen Content

Glycogen content of liver tissue was measured with Glycogen Assay Kit (Biovision, USA) according to the manual's instructions.

### 4.8. Glucose Production Assay

Glucose production of liver tissue was measured using a Glucose Oxidase Kit (Applygen Technologies Inc.) according to the manual's instructions.

### 4.9. Western Blot

Total protein of liver tissue was extracted with Protein Extraction Kit (Applygen Technologies Inc). HepG2 cell lysates were prepared using lysis buffer containing 50 mM Tris-HCl (pH 7.5), 1 mM EDTA, 1% Triton X-100, protease and phosphatase inhibitor, 0.1 mM PMSF, and 1 *μ*g/mL leupeptin. Harvested lysates were centrifuged at 10,000 ×g for 10 min at 4°C. 50 *μ*g protein was subjected to 10% SDS-PAGE and then transferred to the PVDF membranes (Pall Corporation, Pensacola). We used primary antibodies: Nrf2, SOD2, CAT, GSK3*β* (pSer9), GSK3*β*, beta-actin (Santa Cruz Biotechnology), IRS-1 (pSer307), IRS-1, PI3K (pTyr458/199), PI3K, AKT (pSer473), AKT, JNK (pThr183/Tyr), JNK, HO-1 (Cell Signaling Technology), and Nrf2 (pS40) (Abcam). We used secondary antibodies: anti-rabbit antibody, anti-mouse antibody, and anti-goat antibody. Immunoblotting was detected with enhanced chemiluminescence (Pierce Biotechnology, USA). Densities of bands were determined with scanner (Epson Perfection V33).

### 4.10. Immunofluorescence Staining

HepG2 cells were planted on coverslips and incubated overnight. Cells were fixed in 4% paraformaldehyde for 10 min at room temperature and washed with cold PBS. And cells were treated with 0.1% Triton X-100 for 10 min. Cells were incubated for 1 hr with anti-pNrf2 (1 : 100) at room temperature and washed with cold PBS. Cells were incubated for 1 hr with FITC-conjugated goat anti-rabbit IgG (1 : 200) in the dark at room temperature and washed twice with cold PBS. The nuclei were stained with DAPI (5 mg/mL) (Beyotime, China) for 5 min in the dark. Finally, coverslips were observed under a fluorescence microscope (Olympus IX71).

### 4.11. Detection of Intracellular ROS Generation

For the detection of hepatic superoxide production, an oxidative fluorescent dye dihydroethidium (DHE) was used to evaluate the* in situ* production of superoxides. The 10 *μ*m thick liver frozen sections were incubated for 1 hr with 50 *μ*M DHE (Beyotime, China). Fluorescent signals were scanned using a fluorescence microscopy.

### 4.12. RNA Isolation and Quantitative Real-Time PCR

Total RNA was extracted from liver tissue using Trizol reagent (Invitrogen). RNA concentrations were determined by SmartSpecTM Plus (BIO-RAD, USA). 1 *μ*g of total RNA was transcribed to cDNA using the superscript first-strand synthesis kit (Thermo) following instructions. Real-time PCR analysis was performed with a LightCycler instrument (Roche Applied Science) and SYBR green detection of amplified products. Primers for GCK, PK, PEPCK, G6Pase, IL-6, TNF-*α*, and MCP-1 [46] were previously described. PCR reactions were performed in triplicate in 96-well plates and normalized to house-keeping genes (GAPDH) using the 2^−ΔΔCt^ method.

### 4.13. Statistical Analysis

All values were obtained as means ± SEM. Statistical analysis was performed using the ANOVA multiple comparison test. *P* value <0.05 was considered statistically significant.

## Figures and Tables

**Table 1:** Serum and liver characteristics.

Parameters	ND	HFD	LBPH
Serum glucose (mmol)	5.1 ± 1.3	10.2 ± 1.6	6.7 ± 0.7^a^
Serum insulin (pmol/L)	50.8 ± 1.8	121.4 ± 5.2	70.3 ± 2.6^b^
Serum pyruvate (ng/mL)	2.5 ± 0.5	1.1 ± 0.4	3.4 ± 0.8^a^

Liver SOD (U/mg)	141 ± 19	87 ± 8	196 ± 15^b^
Liver CAT (U/mg)	11.5 ± 1.1	5.8 ± 0.9	19.4 ± 1.2^b^
Liver GSH (*μ*mol/L)	495 ± 36	352 ± 27	630 ± 58^c^
Liver GSSG (*μ*mol/L)	46.2 ± 6.5	67.0 ± 8.5	43.2 ± 6.9^a^

ND, normal diet group; HFD, high-fat diet group; LBPH, (100 mg/kg) LBP plus high-fat diet group. Values are performed as means ± SEM (*n* = 6–8 per group), ^a^ *P* < 0.05, ^b^ *P* < 0.01, and ^c^ *P* < 0.001 versus HFD.

## Abbreviations

LBP: * Lycium barbarum* polysaccharides
CAT: Catalase
GCK: Gluconokinase
G6Pase: Glucose-6-phosphatase
GSH: Glutathione
GSK3*β*: Glycogen synthase kinase 3*β*
HFD: High-fat diet
HO-1: Heme oxygenase 1
IRS-1: Insulin receptor substrate-1
JNK: Jun N-terminal kinases
MCP: Monocyte chemotactic protein
ND: Normal diet
Nrf2: NF-E2-related factor 2
ROS: Reactive oxygen species
PEPCK: Phosphoenolpyruvate carboxykinase
PK: Pyruvate kinase
PI3K: Phosphatidylinositol 3-kinase
SOD2: Superoxide dismutase 2
TNF-*α*: Tumor necrosis factor alpha.
